# Economic burden of psoriasis in Southern Iran in 2022

**DOI:** 10.1186/s13690-024-01332-2

**Published:** 2024-07-08

**Authors:** Ramin Ravangard, Moslem Mirzaei, Mehdi Ghahartars, Abdosaleh Jafari

**Affiliations:** 1 Health Human Resources Research Centre, School of Health Management and Information Sciences, Shiraz University of Medical Sciences, Shiraz, Iran; 2 Student Research Committee, Shiraz University of Medical Sciences, Shiraz, Iran; 3 Molecular Dermatology Research Center, Shahid Faghihi Hospital, Shiraz University of Medical Sciences, Shiraz, Iran; 4 Department of Dermatology, School of Medicine, Shiraz University of Medical Sciences, Shiraz, Iran

**Keywords:** Psoriasis, Economic burden, Direct medical costs, Direct non-medical costs, Indirect costs

## Abstract

**Introduction:**

Psoriasis is a common, chronic, and non-contagious skin disease that has no known cause or treatment. Various medical costs for skin disorders, including psoriasis, can be expensive and lifelong. The purpose of the present study was to determine the economic burden of psoriasis in patients admitted to general hospitals affiliated with Shiraz University of Medical Sciences, Iran in 2022.

**Materials and methods:**

This research was a descriptive, cross-sectional, cost of illness study from a societal perspective. All psoriasis patients (*N* = 118) admitted to the hospitals affiliated with Shiraz University of Medical Sciences in 2022 were examined. 7 participants refused to cooperate and were excluded from the study and, the information of 111 patients was collected. A researcher-made data collection form was used to collect data. A prevalence-based approach was used to prepare cost data, and the costing approach was bottom-up. The productivity lost due to the absenteeism of patients and their companions was estimated using the human capital approach. Microsoft Excel ® 2016 was applied to analyze the data.

**Results:**

The mean annual cost per psoriasis patient was estimated to be US$ 30,374.21. Its highest and lowest share was related to direct medical costs (88.61%), direct non-medical costs (7.3%) and indirect costs (4.09%), respectively. Also, the highest mean direct medical, direct non-medical, and indirect costs per patient were related to those of medicine (93.11%), transportation (51.65%), and absenteeism of the patients’ companions due to patient care (71.73%).

**Conclusion:**

Considering that the major contributor in the direct medical cost of treating psoriasis patients was related to medicine, designing appropriate mechanisms for insurance coverage, and allocating government subsidies for the purchase of medicine, are suggested. The result of the current study has important implications for policymakers in developing guidelines for early diagnosis of this disease and reducing the health economic burden.

**Table:** 

Text box 1. Contributions to the literature
• Psoriasis is a chronic dermatology disease that imposes a significant economic burden on patients and the healthcare system due to direct and indirect costs. Early diagnosis of this disease can help to reduce the health economic burden.
• Based on the results of the present study, the mean annual cost per patient was US$ 30,374.21, and the largest share was related to medication. Also, the highest direct medical, direct non-medical, and indirect costs were related to those of medicine, transportation, and absenteeism of the patients’ companions due to patient care.
• According to the findings and to reduce costs imposed on patients, designing appropriate mechanisms for insurance coverage, allocating government subsidies for the purchase of medicine, improving the appointment status, providing telemedicine services, increasing accommodation for the patients’ companions, and providing public training to make the society aware of the early symptoms of psoriasis are suggested.

## Introduction

Psoriasis is a common, chronic, and non-contagious skin disease [1] and often appears in the form of plaque, guttate, erythrodermic, pustular, reverse, sebopsoriasis, and psoriatic arthritis [2, 3]. Different types of skin lesions appear in psoriasis patients, but all have the same important symptoms, including erythema, thickening, and scaling. In severe psoriasis, the lesions are often itchy [4]. The diagnosis of this disease is usually based on the characteristics found in the skin examination. However, if some symptoms of the patients are not typical, a skin biopsy may be needed to confirm the diagnosis. Mild psoriasis is treated with treatments that are applied directly to the skin, and moderate to severe psoriasis is usually treated with systemic and biological drugs that are injected into the subcutaneous or intravenous route [5].

According to the Global Burden of Disease Study (2019), the prevalence of psoriasis in the worldwide was 40,805,386 people. The global prevalence of this illness in Asia was 38.58%, Europe (29.25%), North America (11.5%), Latin America and the Caribbean (8.48%), North Africa and Southwest Asia (5.95%), South Africa (4.92%), and Oceania (1.32%) [6]. The prevalence of psoriasis in 2019 in Iran’s neighboring countries, including Turkmenistan, Azerbaijan, Armenia, and Saudi Arabia, was between 0.5 and 1%, and in Pakistan, Afghanistan, Turkey, Iraq, Kuwait, Qatar, the United Arab Emirates, and Oman, it was below 0.5%. The prevalence of this disease in Iran in 2019 was 0.45% (373,112 people), such that its prevalence was 0.57% (472,609 adults) in adults and 0.06% in children (49,748 children) [7, 8].

The annual direct and indirect cost of psoriasis in the United States was about $112 billion in 2013 [9]. The estimated total annual cost of moderate to severe plaque psoriasis in Brazil was US$ 758,467, with a mean of US$4,034 per patient in 2016 [10]. Also, in Sweden in 2016, the mean direct and indirect costs in psoriasis patients were estimated to be US$ 1,365 (62%) and US$ 3,319 (50%) higher than the control group, respectively [11]. In Malaysia, in 2017, the cost of each admitted patient per day was US$ 316, and the cost of each outpatient patient was US$ 85 [12]. The mean total 1-year medical costs of psoriasis outpatients in Iran in 2020 was US$ 785, of which the share of direct medical, direct non-medical, and indirect costs was 78.2%, 12.3%, and 9.5%, respectively, and the highest direct medical, direct non-medical and indirect costs were related to medicine, transportation of patients and their companions, and patient’s absenteeism from work due to illness [13]. In Iran in 2021, 35% of patients with psoriasis spent less than US$ 25 per month, 18.2% between 25 and US$ 50, 13.5% between US$ 50 and US$ 75, and 33.3% more than US$ 75 for treatment [14].

Cost of illness analysis was the first economic evaluation technique used in the health field [15, 16, 17]; its basic purpose is to evaluate the economic burden that the disease imposes on society [18]. This type of analysis measures the degree of diseases or risk factors in terms of economic burden and shows the problems in terms of currency units [19]. In cost of illness analysis, researchers are required to identify, list, measure, and value the costs that a disease and its associated diseases can cause [20, 21].

Since the researchers could not find a comprehensive study related to the economic burden imposed on admitted patients with psoriasis in Iran, in this study, the economic burden of psoriasis in patients admitted to general hospitals affiliated with Shiraz University of Medical Sciences, Iran in 2022 was examined.

## Methods

This research was a descriptive, cross-sectional, cost of illness study from a societal perspective. In this study, all psoriasis patients (*N* = 118) who were admitted to the general hospitals of Shiraz University of Medical Sciences, Iran in 2022 were examined. 7 participants refused to cooperate and were excluded from the study and, the information of 111 patients was collected.

In this study, to determine the economic burden of psoriasis in admitted patients of Shiraz University of Medical Sciences, the societal perspective was used (all costs covered by the patient and third-party payers), which is the most complete perspective because it includes all direct medical costs, direct non-medical costs, and indirect costs for all members of society [22].

A prevalence-based approach was used to prepare cost data. This approach measures the economic burden of a disease in a certain period, which is usually 1 year [18]. Also, the costing approach was bottom-up (collecting information using patient-level data). Compared to the top-down approach, this approach provides more detailed data about the cost of the disease and the use of resources allocated to health [23].

Each US$ was considered equivalent to 42,000 Rials based on the exchange rate announced by the Central Bank of the Islamic Republic of Iran in 2022 [24].

In order to collect data, a researcher-made data collection form was prepared and used based on the objectives of the study, a review of previous studies in this field, and consultations with specialists in skin diseases and health economics.

The first part of the data collection form contains questions related to demographic and clinical characteristics of the patient, including gender, age, age of disease onset, duration of the disease, accommodation status, marital status, education level, role of the patient in the family, employment status, the patient income, insurance status, disease symptoms, and smoking status were collected through the self-report of patients or their companions and their medical records. Except for marital status, education level, patient’s income, insurance status, disease symptoms, and questions about transportation and accommodation related to direct non-medical expenses, the rest of the questions were asked as open questions.

The next part of this form was related to direct medical costs, including the costs related to medicine, hoteling, laboratory services, visits,^1^ nursing care package, companion and baby hospital beds, department consumables, common nursing consumable services, and other services, which was often received from patients’ financial bills. Direct medical costs included the total costs paid by the patient and the insurance organization.

Another part of the data in this form was related to direct non-medical costs, including questions about the cost of transportation of patients and their companions, the cost of accommodation and food for patients and their companions, the cost of changes in the home environment to adapt to the disease, the cost of care and nursing of the patient at home, the costs related to the special diet of this disease and the parking cost. This type of cost was received by the self-reporting of the patients or their companions through a telephone call.

The final part of the data collection form was related to indirect costs including patient’s absenteeism from work due to illness and their companions due to patient care. In order to receive this type of cost, the patients and their companions were either directly asked how much of their income they lost or, according to the number of days they were off work and the income of those days (reported by the patients or their caregivers); this amount was calculated by the researcher.

Data related to direct medical costs were often extracted from the medical records of patients, and data related to direct non-medical costs and indirect costs were obtained by asking the patients or their companions. To reduce the recall error, information about direct non-medical costs and indirect costs was asked from the patients for 3 months, and according to the frequent visits of patients for follow-up treatment, the costs were estimated for 1 year.

Productivity lost due to the absenteeism of patients and their companions was estimated using the human capital approach. The human capital method considers the patient’s perspective and counts every hour that they did not work as a lost hour [25]. For those patients who refused to provide their income, as well as for housewives and students, the minimum daily wage set by the Ministry of Cooperatives, Labor and Social Welfare in 2022 was used as proxy (the mean daily wage was US$ 33.17) [26]. Given that none of the patients died of psoriasis during the study period, this study did not have any cost of life years lost due to premature death.

Microsoft Excel ® 2016 was used to analyze the data [27].

## Results

Based on the results of the data collected from 111 patients participating in the study, most of them were male (53.2%), 30 to 50 years old (52.3%), with a disease duration of less than 10 years (50.5%), and with the onset of the disease under 30 years (53.2%). Also, most of the patients were living in Shiraz (55.9%), married (81.1%), with education below diploma (40.5%), head of household (50.5%), unemployed, student or housewife (56.8%), with no income (56.8%), covered by social security insurance (46.8%), with plaque and scaling (82.9%), and non-smoker (75.7%) (Table 1).

**Table 1:** Demographic and clinical characteristics of psoriasis patients admitted to Shiraz University of Medical Sciences in 2022 (*N* = 111)

Specifications		Frequency (%)	Specifications			Frequency (%)
Gender	Man	59 (53.2%)	The role of the patient in the family	Head of family	Yes	56 (50.5%)
	Woman	52 (46.8%)			No	55 (49.5%)
Age	< 30	18 (16.2%)	Employment status	Unemployed, student, or housewife		63 (56.8%)
	30–50	58 (52.3%)		Employee or retired		17 (15.3%)
	50 <	35 (31.5%)		Self-employed		31 (27.9%)
Age of disease onset	< 30	59 (53.2%)		No income		63 (56.8%)
	30–50	33 (29.7%)	Patient income	≤ US$ 1200		18 (16.2%)
	50 <	1 9(17.1%)		> 1200 US$		30 (27.0%)
				No income		63(56.8%)
Duration of the disease	< 10	56 (50.5%)	Insurance status	No insurance		4 (3.6%)
	10–20	37 (33.3%)		Social security		52 (46.9%)
	20 <	18 (16.2%)		Iran health insurance		47 (42.3%)
Place of residence	Shiraz	62 (55.9%)		Medical services of the armed forces		8 (7.2%)
	Other cities except Shiraz	49 (44.1%)	Symptoms of the patients^a^	Plaque and scaling		92 (82.9%)
Education level	Illiterate	13 (11.7%)		Itching		90 (81.1%)
	Below diploma	45 (40.6%)		Swelling		60 (54.1%)
	Diploma	28 (25.2%)		Fever		37 (33.3%)
	Academic degrees (Bachelor’s degree or post graduate)	25 (22.5%)	Smoking status	Smoking		27 (24.3%)
Marital status	Single	21 (18.9%)		Non-smoking		84 (75.7%)
	Married	90 (81.1%)				

^a^Patients can have more than one symptom and that is why it does not add up to 100%

Based on the results obtained, the mean annual cost per patient was US$ 30,374.21; its highest and lowest shares were related to direct medical costs (88.61%) and indirect costs (4.09%), respectively. The highest mean direct medical, direct non-medical, and indirect costs per patient were related to those of medicine (93.11%), transportation of patients and their companions (51.65%), and absenteeism of the patients’ companions due to patient care (71.73%), respectively (Table 2).

**Table 2:** The mean annual cost of direct medical, direct non-medical, and indirect medical per psoriasis patient admitted to Shiraz University of Medical Sciences in 2022 (*N* = 111)

Type of costs			Mean (US$)	Standard deviation (US$)	%	% of total costs
Direct medical costs	Medicine	Infliximab injection ($15,417.02)	25,060.18	16,906.42	93.11	88.61
		Remicade injection ($7182.24)				
		Other ($2,460.92)				
	Hoteling^a^		523.58	706.25	1.95	
	Laboratory services		488.14	524.67	1.81	
	Visit^b^		401.16	333.57	1.49	
	Other services		135.70	120.73	0.50	
	Nursing care package		131.67	163.59	0.49	
	Companion and baby hospital bed		84.51	112.66	0.31	
	Department consumables		58.29	45.76	0.22	
	Common nursing consumable services		31.41	42.37	0.12	
	Total		26,914.61	18,956.01	100	
Direct non-medical costs	Transportation of patients and their companions		1,145.33	1,705.31	51.65	7.30
	The changes in the home environment to adapt to the disease		373.23	2,373.50	16.83	
	Food for patients and their companions		292.56	558.00	13.19	
	The accommodation of patients and their companions^c^		253.80	1,073.64	11.44	
	The care and nursing of the patient at home		111.33	746.79	5.02	
	A special diet of disease		33.33	169.12	1.50	
	Parking^d^		7.98	25.57	0.36	
	Total		2,217.55	6,651.93	100	
Indirect costs	The absenteeism of the patients’ companions due to patient care		890.92	3,589.52	71.73	4.09
	The patient’s absenteeism due to illness		351.13	400.12	28.27	
	Total		1,242.05	3,989.64	100	
Total costs			30,374.21	29,597.57	100	100

^a^The fixed fees paid to the hospital for staying there

^b^The visit fee consists of two parts:

1- 4.56 average annual outpatient visits per patient for $11.73

2- 7.74 average annual hospitalization visits per patient for $44.92

^c^Considering that the present study was carried out from a societal perspective, the cost accommodation of patients and their companions were calculated

^d^Parking outside of hospital

In addition, the average total costs for men and women were US$ 4,860.70 (SD = US$ 3,075.75) and US$ 4,999.67 (SD = US$ 2,836.57), respectively. The average total costs for young people (Age < 30), middle-aged people (30 ≤ Age ≤ 50), and for older people (Age > 50) were US$ 5,544.31 (SD = US$ 3,018.60), US$ 5,018.37 (SD = US$ 3,178.71) and US$ 4,454.39 (SD = US$ 2,506.31), respectively.

## Discussion

The purpose of the present study was to determine the economic burden of psoriasis in patients admitted to general hospitals affiliated with Shiraz University of Medical Sciences, Iran in 2022.

The results showed that the largest share of total costs was related to direct medical costs. The results of Min et al. (2023), Zarei et al. (2021), Lopes et al. (2019), and Steinke et al. (2013) were consistent with this study [10, 13, 28, 29], but in Moradi (2017), the highest amount was related to indirect costs [30]. One possible reason is that in the present study, only the cost of absenteeism of patients and their companions was calculated as productivity lost, while in Moradi’s study in Iran (2017), the reduction of productivity due to presenteeism was also estimated. In Moradi’s study, the presenteeism was responsible for 45% of the total per patient annual cost of psoriasis in Iran. Another possible reason was due to the large number of unemployed, student, and housewife patients in the present study.

On the other hand, the lowest share of total costs was related to indirect costs. The result of Zarei et al. (2019) was consistent with this study [13], but in Min et al. (2023), Lopes et al. (2019), Moradi (2017), and Steinke et al. (2013), the lowest amount was related to direct non-medical costs [10, 28–30]. One possible reason for the lower indirect costs compared to direct non-medical costs in this study was the low level of productivity lost due to the absenteeism of patients and their companions due to their low income.

The largest share of direct medical costs was related to medicine. The results of Löfvendahl et al. (2022), Zarei et al. (2021), Amiri et al. (2021), Lopes et al. (2019), Azizam et al. (2019) and Levy et al. (2012) were also consistent with this study [10, 12, 13, 31–33]. The high share of medicine was due to its high price, especially the price of biological drugs [28, 34] and lack of government and insurance support. It can be said that, because psoriasis is an autoimmune disease and has a long treatment period [4], and also, to prevent the recurrence of the disease, the patients had to use medicine continuously. In order to reduce the direct treatment costs imposed on patients due to hospitalization in private centers for drug injections, it is expected that the medical authorities will increase the number of beds in the dermatology department in government medical centers to solve the problem of the limitation of patient admission capacity.

Direct non-medical costs were only a very small part of the total costs of psoriasis, although transportation of patients and their companions accounted for the largest share of direct non-medical costs. The results of Zarei et al. (2021) and Lopes et al. (2019) were also indicated that transportation costs were responsible for the highest share of direct non-medical costs [10, 13]. Unlike the cost of transportation, which is necessary for all patients, other direct non-medical costs, such as the cost of food and accommodation, can be greatly reduced by using alternative strategies. For example, patients and their companions used to sleep in tents, use the patient’s companion accommodation, bring food from home, and give up the expensive diet suitable for the disease. Also, more than half of the patients were living in Shiraz, and they usually did not pay for accommodation and food, but they had to pay for transportation [35].

In order to reduce non-medical direct costs such as transportation, food and accommodation and indirect costs including lost productivity costs due to the absenteeism of patients and their companions, it is necessary to provide the necessary infrastructure to provide teledermatology services to patients who have to long travel to receive medical services or travel is difficult for them due to severe lesions caused by psoriasis.

The limitations of this study include the non-cooperation of some patients or their companions to provide the required data (7 patients), forgetting the exact amount of some costs, and providing incomplete data by patients or their companions. Also, indirect cost and direct non-medical cost were estimated based on self-reports of patients or their companions.

## Conclusion

The mean annual cost per patient was US$ 30,374.21, and the largest share was related to direct medical costs and medications. Also, the highest direct medical, direct non-medical, and indirect costs were related to those of medicine, transportation, and absenteeism of the patients’ companions due to patient care.

Considering that the major contributor in the direct medical cost of treating psoriasis patients was related to medicine, designing appropriate mechanisms for insurance coverage, and allocating government subsidies for the purchase of medicine, are suggested.

## Data Availability

No datasets were generated or analysed during the current study.

## Footnotes

1 The cost of visit was explained in the footnote of Table 2
